# Seroprevalence of viral hepatitis B and C infections among healthcare workers in Ethiopia: A systematic review and meta-analysis

**DOI:** 10.1371/journal.pone.0312959

**Published:** 2024-11-07

**Authors:** Getu Girmay, Gezahegn Bewket, Azanaw Amare, Abiy Ayele Angelo, Yenesew Mihret Wondmagegn, Abebaw Setegn, Menberu Wubete, Muluneh Assefa

**Affiliations:** 1 Department of Immunology and Molecular Biology, School of Biomedical and Laboratory Science, College of Medicine and Health Sciences, University of Gondar, Gondar, Ethiopia; 2 Department of Medical Microbiology, School of Biomedical and Laboratory Science, College of Medicine and Health Sciences, University of Gondar, Gondar, Ethiopia; 3 Department of Medical Parasitology, School of Biomedical and Laboratory Science, College of Medicine and Health Sciences, University of Gondar, Gondar, Ethiopia; 4 Department of Medical Laboratory sciences, College of Medicine and Health Sciences, Dire Dawa University, Dire Dawa, Ethiopia; FIOCRUZ, BRAZIL

## Abstract

**Background:**

Healthcare workers (HCWs) are at higher risk of contracting hepatitis B virus (HBV) and hepatitis C virus (HCV) infections. Currently, there is no estimate of pooled data on the prevalence of HBV and HCV infections among HCWs in the country. Thus, this review aimed to determine the pooled prevalence of hepatitis B and C infections among HCWs in Ethiopia.

**Materials and methods:**

A comprehensive literature search was conducted using electronic databases, including PubMed, Cochrane Library, Science Direct, Hinari, and African Journals Online to identify pertinent articles from the inception to April 2024. The protocol has been registered in the International Prospective Register of Systematic Reviews (PROSPERO; CRD42024527940) and conducted per the Preferred Reporting Items for Systematic Reviews and Meta-Analyses (PRISMA) guidelines. Data were extracted independently by two authors and analyzed using STATA version 11 software. A random-effect model and Egger’s test were computed to estimate the pooled prevalence and assess publication bias, respectively.

**Results:**

A total of 18 studies involving4,948 healthcare workers were included in this review to estimate the pooled prevalence of HBV and HCV infections among HCWs in Ethiopia. The overall prevalence of HBV was 5.93% (95% CI; 3.22–8.63). The sub-group analysis showed that the prevalence of HBV among medical waste handlers and health professionals was8.6% (95% CI; 3.01–14.13) and 4.98% (95% CI; 1.85–8.11), respectively. The combined prevalence of HCV was 1.12% (95% CI; -4.19–6.43). In the sub-group analysis, the prevalence of HCV among medical waste handlers and health professionals was1.44% (95% CI; -5.28–8.18) and 0.59% (95% CI; -8.09–9.27), respectively.

**Conclusion:**

In this review, we found a higher (5.93%) and moderate (1.12%) prevalence of HBV and HCV infections, respectively among Ethiopian HCWs. Therefore, to reduce the infectious burden of HBV and HCV among HCWs; there is a need to strict adherence to infection prevention and control measures. In addition, adequate HBV vaccination coverage for HCWs is mandatory to reduce the burden of HBV infection in the country.

## Introduction

Hepatitis B virus (HBV) and hepatitis C virus (HCV) are the two most prevalent types of viral hepatitis and are common causes of cirrhosis, hepatocellular carcinoma, and chronic hepatitis infection in developing countries [1, 2].Viral hepatitis caused by hepatitis B and C viruses is the sixth-leading cause of morbidity and mortality worldwide. In 2016, around 292 million individuals were estimated to have had a chronic HBV infection, and 71.1 million to have had a chronic HCV infection [3, 4]. About 70 million individuals in Africa suffer from chronic viral hepatitis; 60 million of these cases are caused by HBV and 10 million by HCV infections. Ethiopia is also considered to have a medium to high prevalence of viral hepatitis [5, 6].

Healthcare workers (HCWs) are more likely to be exposed to HBV and HCV infections. Those HCWs, particularly Health professionals and medical waste handlers have a four-fold increased risk of contracting HBV and HCV infections compared to the general public who do not work in healthcare settings [7, 8]. Healthcare workers are commonly at risk of acquiring HBV and HCV infections by percutaneous exposure to contaminated sharp instruments or mucosal-cutaneous exposure to possibly infectious blood or blood products [7, 9]. Based on the World Health Organization (WHO) report, from a total of 36 million HCWs across the world; about 3 million of them could encounter sharp and needle stick injuries annually. Thus, around two and one million HCWs have been exposed to the risk of HBV and HCV infections, respectively [9, 10]. In addition, nearly half of African HCWs are occupationally exposed to blood products and body fluids, thus appears to be an increased incidence of hepatitis B and C viruses among HCWs in the continent [11, 12].

Assessing the relative contributions of HBV and HCV to the prevalence of liver disease is essential for setting public health strategies and strengthening preventative measures [13, 14]. Despite, the WHO’s recommendations for the hepatitis B vaccine, the vaccination coverage among HCWs in the country is insufficient as evidenced by a meta-analysis report only 20% of Ethiopian HCWs had received a full-dose HBV vaccine [15, 16]. Several studies have been conducted in Ethiopia on the prevalence of HBV and HCV infections with varying results [17–21] and the pooled prevalence is still uncertain. For instance, the prevalence of HBV was 2.5% in southwest Ethiopia [17], 6.3% in Addis Ababa [22], and 9.6% in eastern Ethiopia [23]. Besides, the prevalence of HCV was 0.42% in southwest Ethiopia [17] and 1.59% in Addis Ababa [18]. Currently, no estimate of pooled data shows the prevalence of HBV and HCV among HCWs in the country. Thus, this review aimed to determine the pooled prevalence of hepatitis B and C infections among HCWs in Ethiopia.

## Methods

### Study design and protocol registration

This systematic review and meta-analysis were performed per the Preferred Reporting Items for Systematic Reviews and Meta-Analyses (PRISMA) guidelines [24] (**S1 Table**). The protocol has been registered in the International Prospective Register of Systematic Reviews (PROSPERO), with a reference number; CRD42024527940.

### Search strategy

A comprehensive systematic literature search was carried out using electronic databases, including PubMed, Cochrane Library, Science Direct, Hinari, and African Journals Online, to find all relevant articles reporting the prevalence of HBV and HCV viruses among HCWs in Ethiopia from the inception to April 2024. Additional searches were also conducted to find relevant studies using Google Scholar, Google, manual searching, and cross-references of identified primary studies and/or review articles. We applied the search terms separately and in combination with Boolean operators such as "OR" and "AND". The following search strategy has been developed for PubMed and then customized for other electronic databases: (((((((((((Seroprevalence) OR (Prevalence)) AND ("hepatitis B virus")) OR ("HBV")) OR ("hepatitis C virus")) OR ("HCV"))) OR (("hepatitis viruses"))) AND (healthcare workers)) OR (health professional)) OR (healthcare provider)) OR (medical waste handler) AND (Ethiopia [MeSH]).

### Eligibility criteria

#### Inclusion criteria

We included studies based on the condition/context/population (CoCoP) approach. Healthcare workers (P) are defined in this review as physicians, clinical nurses, medical laboratory professionals, midwives, medical assistants, and medical waste handlers/collectors who have direct contact with patient body fluids, including blood, sperm, and stool samples, as well as exposure to sharp objects and needle stick injuries. Studies conducted in Ethiopia (Co) that are cross-sectional, case-control, and cohort in nature reported the seroprevalence of HBV and/or HCV infection (Co), with no restriction in the year of publication. Studies using the hepatitis B surface antigen (HBsAg) and anti-HCV antibody for HBV and HCV diagnosis respectively were included in this review.

#### Exclusion criteria

Studies that did not report the prevalence of HBV and/or HCV, as well as case reports, reviews, editorial letters, case series, and poster presentations, were included. This review also excluded studies that were conducted outside of Ethiopia.

### Study selection and quality assessment

All potentially suitable papers were examined following duplicates or ineligible papers were removed. Full-text articles were reviewed per the eligibility criteria to identify studies for inclusion or exclusion in the current study. Relevant information was extracted from all eligible studies by two independent review authors (GG and MA) and discrepancies between the authors were addressed through discussion with the third review author (AA). The Joana Briggs Institute (JBI) critical appraisal checklist for simple prevalence studies was used to evaluate the quality of the included studies [25]. The JBI checklist is composed of 9 questions. (Q1) Was the sample frame appropriate to address the target population? (Q2) Were study participants sampled appropriately? (Q3) Was the sample size adequate? (Q4) Were the study subjects and the setting described in detail? (Q5) Was the data analysis conducted with sufficient coverage of the identified sample? (Q6)Were valid methods used for the identification of the condition? (Q7) Was the condition measured in a standard, reliable way for all participants? (Q8) Was there an appropriate statistical analysis? (Q9) Was the response rate adequate, and if not, was the low response rate managed appropriately? A score of 1 was given for answering "Yes" to each question, while 0 was given for answering "Not reported" or "Not appropriate." Subsequently, the individual response scores were tallied to arrive at a final score that ranged from 0 to 9.Based on the assigned points, the authors decided to categorize the included articles’ quality as high (7 to 9), moderate (4 to 6), or low (0 to 3) (**S2 Table**).

### Data extraction

Two review authors (GG and MA) independently evaluated the methodological quality of the included studies and extracted all the relevant information; discrepancies were addressed through discussion with a third review author (AA). A standardized data collection form was used to collect information on the publication year, study period, study setting/region, study population, sample size, study design, sampling procedure/method, diagnostic methods used to detect HBsAg or anti-HCV, and HBV vaccination status.

### Statistical methods and analysis

The extraction and entry of all the relevant data were performed using Microsoft Excel and exported to Stata version 11 software for analysis. The pooled prevalence of HBV and/or HCV with 95% confidence intervals was depicted using a forest plot. An index of heterogeneity (I^2^ statistic) was used to assess the degree of heterogeneity among the studies. The level of heterogeneity was interpreted as Low, moderate, and high when the values of the I^2^ statistic became 25%, 50%, and 75%, respectively [26]. A random-effects model was used in all pooled analyses due to the variations in the prevalence of included studies. The pooled prevalence of HBV and/or HCV among different study populations, regions, and other parameters was assessed using the sub-group analysis. The presence of publication bias was demonstrated using a funnel plot and Egger’s test statistics. A p-value < 0.05 in Egger’s test was considered as evidence of significant publication bias.

## Results

### Literature search results

**Fig 1:**
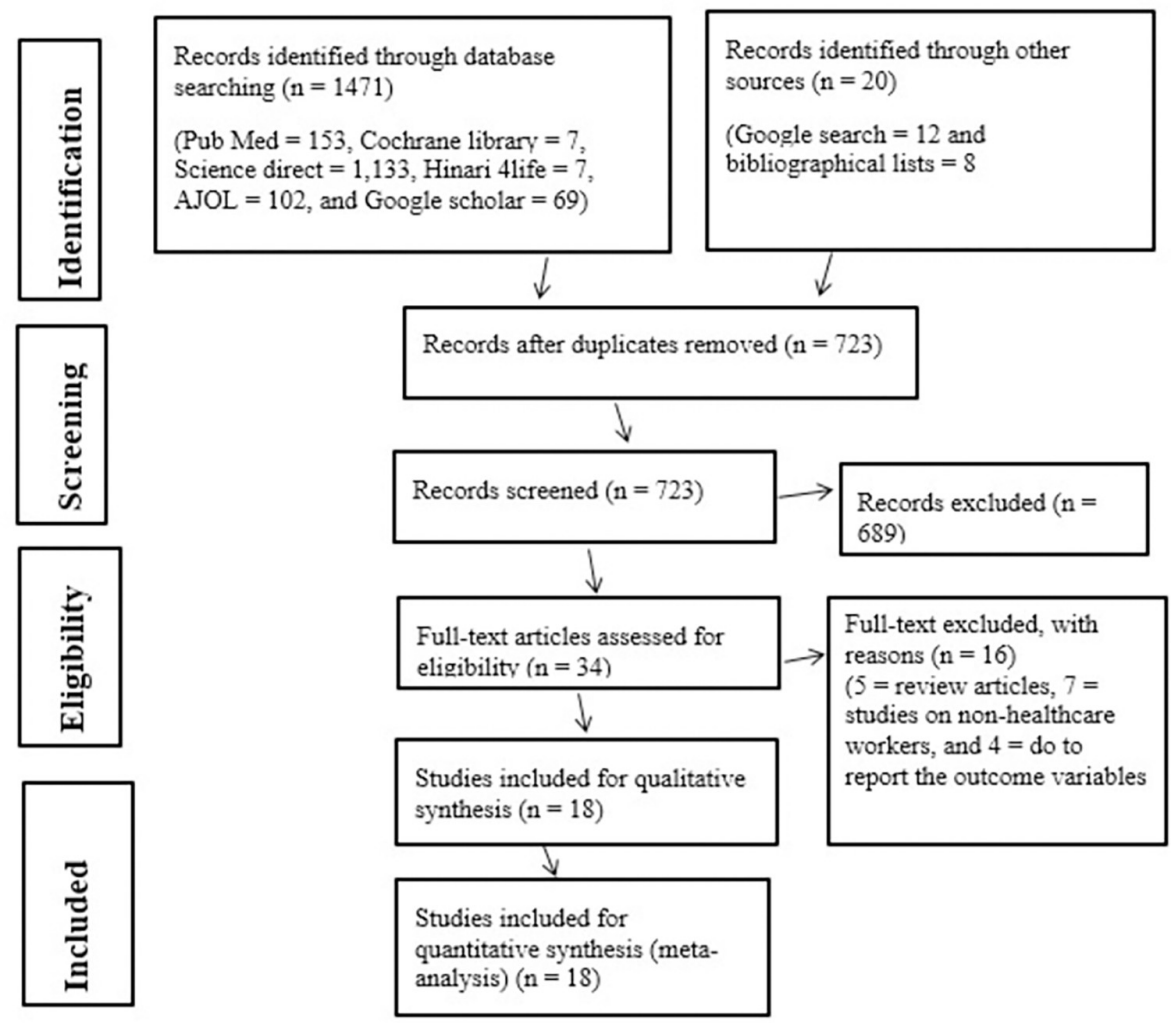
Flow diagram describing the selection of studies for systematic review and meta-analysis on the prevalence of HBV and HCV among healthcare workers in Ethiopia.

A total of 1,491 studies published between December 1989 and July 2023 that were retrieved from initial electronic searches using international databases, Google Scholar, Google search, and manual searching. Seven hundred sixty-eight records were removed due to duplicates, from the screened 723 studies; 689 studies were excluded following title and abstract screening. In addition, 16 studies were excluded following full-text screening. Thus, a total of 18 eligible studies were included in this systematic review and meta-analysis (**Fig 1**).

### Characteristics of the included studies

A total of 18eligible studies [17–23, 27–37] on 4,948 healthcare workers were included in this systematic review and meta-analyses. Of those, 3,723 and 1,225 were health professionals and medical waste handlers, respectively. Fortunately, all the studies used a cross-sectional study design. Of the 18 included studies, 10 studies used a convenient sampling, followed by 5 studies used simple random sampling, and the other 2 and 1 studies used multi-stage sampling and systematic random sampling, respectively. The majorities (12 studies) were conducted between the years 2013 to 2021 whereas; the other 5 studies were conducted between the years 2001 to 2012. On the other hand, only one study was conducted before the year 2000. Of the 18 studies included, 6 studies were conducted in Addis Ababa city administration, 5 in the Amhara region, 4 in the Oromia region, 2 in the Harari region, and 1 in the Sidama region (**Table 1**).The minimum and maximum number of study participants included were 70 in Amhara region [28] and 612 in Oromia region [35], respectively. Of the 4, 948included healthcare workers, 2, 938 of them were females. The mean age of study participants ranged from 25 to 37.6 years. Of the 18 studies, 7 studies reported the prevalence of both HBV and HCV, whereas 11 studies reported HBV alone. Ten and 7 studies determined the seropositivity of HBsAg and/or anti-HCV using the enzyme-linked immunosorbent assay (ELISA) and rapid test kits, respectively. Whereas, only a single study determined HBsAg seropositivity using a chemiluminescent immunoassay (CIA) (**Table 1**).

**Table 1:** Characteristics of studies included in the meta-analysis on the prevalence of hepatitis B and C viruses among healthcare workers in Ethiopia.

Author, year	Study year	Study region	Sample size	Study design	Sampling method	Study population	Diagnostic method	Sex (M)	Age (mean)	HBV cases	HBV vaccinated (full dose)	HCV cases
Desalegn&G/Selassie, 2013 [29]	2010–11	Addis Ababa	254	Cross-sectional	Convenient	Health professional	Rapid test kit	127	35	6	0	
Geberemicheal et al, 2013 [30]	2012	Oromia	110	Cross-sectional	Simple random	Health professional	Rapid test kit	58	28.2	8	2	
Kefenie et al, 1989 [33]	1987	Addis Ababa	438	Cross-sectional	Convenient	Health professional	ELISA	210	30.5	25		
Abate et al, 2022 [23]	2018	Harari	432	Cross-sectional	Multi-stage	Health professional	Rapid test kit	278	28	42	360	
Yizengaw et al, 2018 [37]	2017	Amhara	268	Cross-sectional	Simple random	Health professional	ELISA	147	28.3	7	25	
Akalu et al, 2016 [27]	2013–14	Addis Ababa	313	Cross-sectional	Convenient	Health professional	CIA	102	31	2	5	
Gebremariam et al, 2019 [31]	2015	Amhara	332	Cross-sectional	Convenient	Health professional	ELISA	198	27	15	11	
Yilma et al, 2021 [36]	2019	Oromia	457	Cross-sectional	Simple random	Health professional	ELISA	217	30	8		
Seid et al, 2005 [32]	2005	Addis Ababa	267	Cross-sectional	Convenient	Health professional	ELISA	143	32	26	35	2
Tufa et al, 2016 [35]	2014–15	Oromia	612	Cross-sectional	Convenient	Health professional	Rapid test kit	250	32	37	39	
Hebo et al, 2019 [17]	2015–16	Oromia	240	Cross-sectional	Simple random	Health professional	ELISA	116	25	6		1
Amsalu et al, 2016 [20]	2014–15	Sidama	152	Cross-sectional	Convenient	Medical waste handler	Rapid test kit	16	31.6	2	7	1
Mengiste et al, 2021 [34]	2018	Harari	260	Cross-sectional	Multi-stage	Medical waste handler	Rapid test kit	23	29	53		
Shiferaw et al, 2011 [22]	2010	Addis Ababa	126	Cross-sectional	Systematic random	Medical waste handler	ELISA	40	35.7	8		
Mussa et al, 2022 [19]	2020	Amhara	265	Cross-sectional	Convenient	Medical waste handler	ELISA	57		16		3
Anagaw et al, 2012 [21]	2011	Amhara	100	Cross-sectional	Convenient	Medical waste handler	Rapid test kit	4	34.7	6		1
Ayele et al, 2023 [28]	2021	Amhara	70	Cross-sectional	Simple random	Medical waste handler	ELISA	15	37.6	6		3
Mekonnen et al, 2015 [18]	2014	Addis Ababa	252	Cross-sectional	Convenient	Medical waste handler	ELISA	9	30.5	9	17	4

M: male, HBV: hepatitis B virus, HCV: hepatitis C virus, ELISA: Enzyme-linked immunosorbent assay, CIA: Chemiluminescent Immunoassay.

### Prevalence of HBV and HCV infections among healthcare workers in Ethiopia

**Fig 2:**
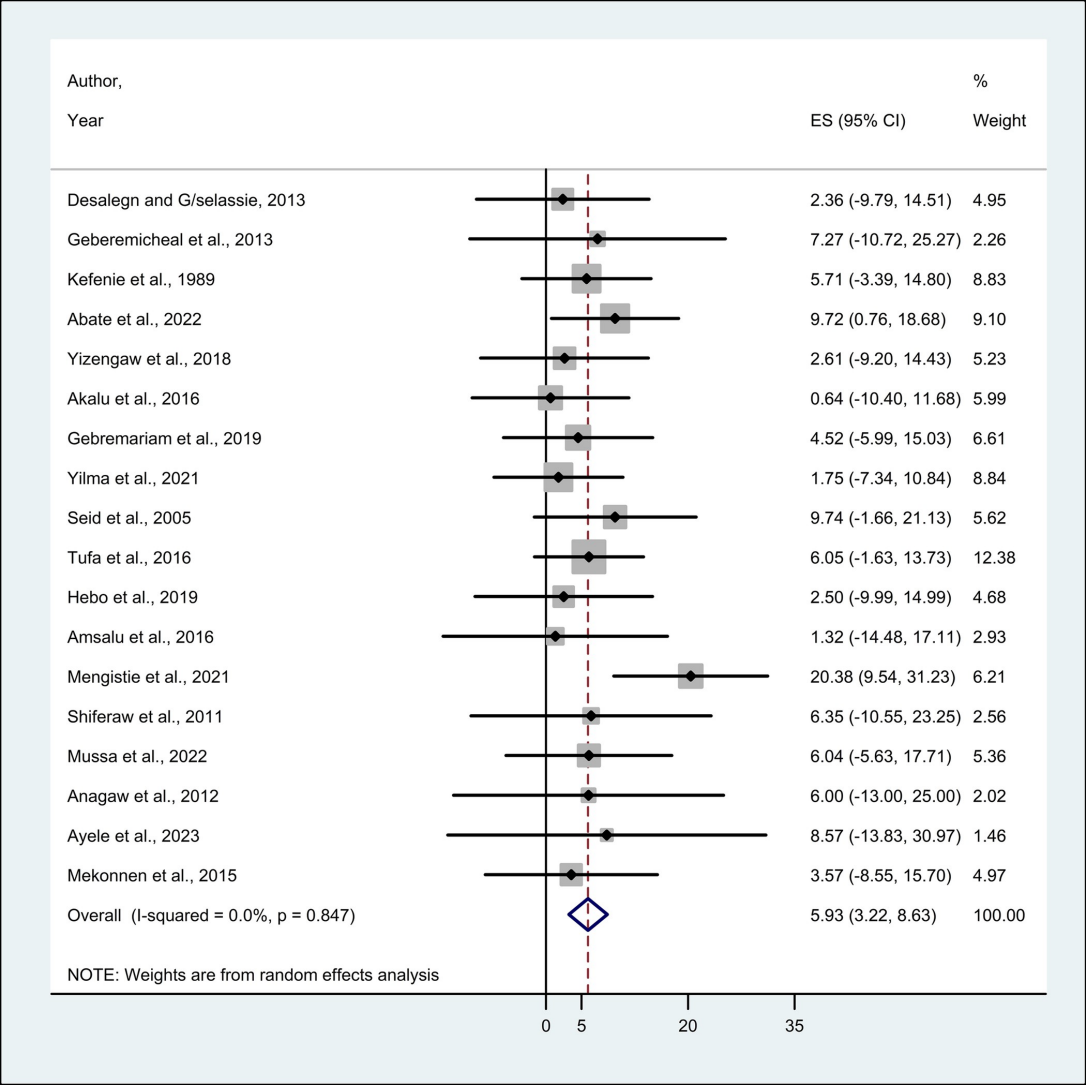
The forest plot showing the pooled prevalence of HBV among healthcare workers in Ethiopia.

**Fig 3:**
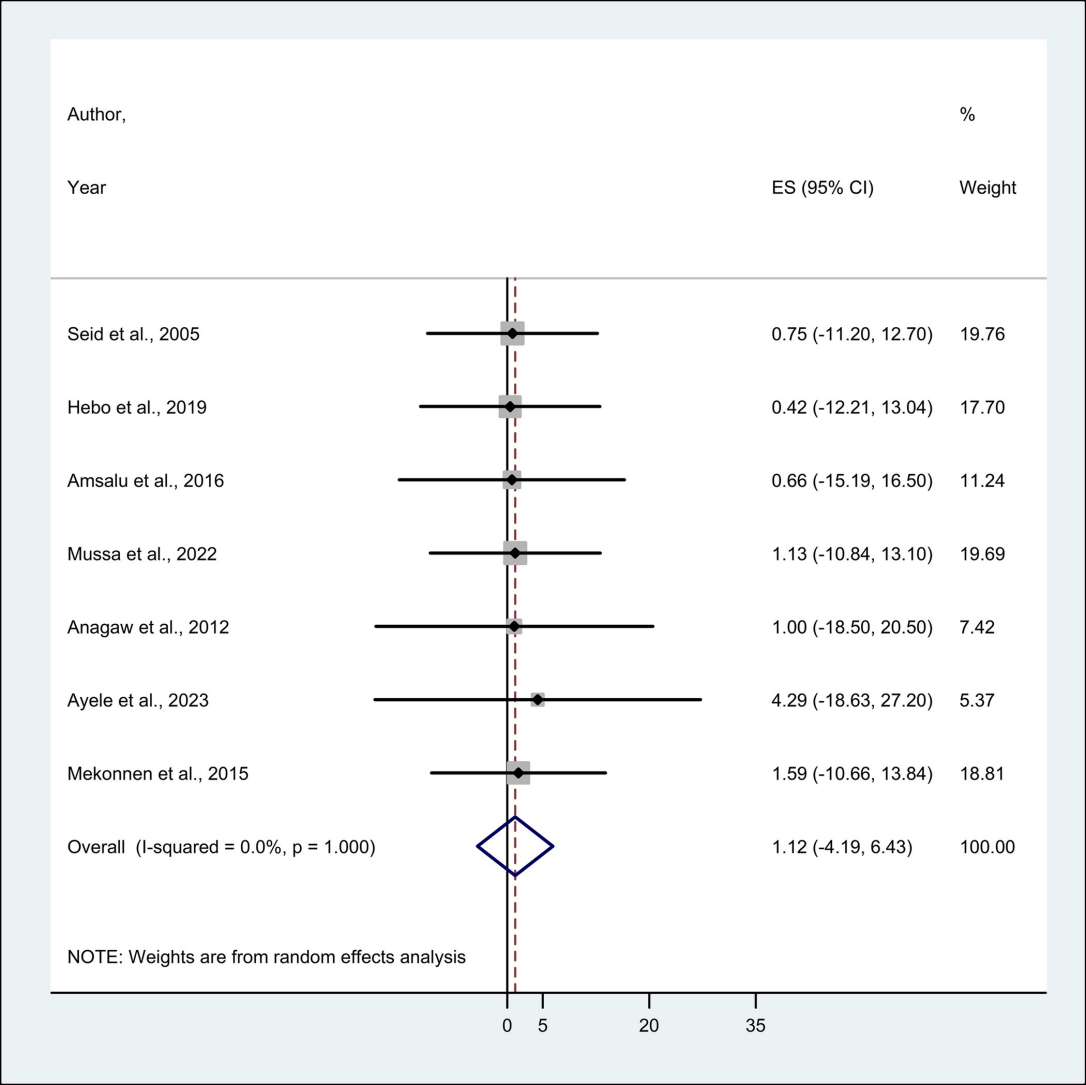
The forest plot showing the pooled prevalence of HCV among healthcare workers in Ethiopia.

This systematic review and meta-analysis included 18 eligible studies to estimate the pooled prevalence of HBV and HCV infection among HCWs. The minimum and maximum prevalence was 0.64% (Addis Ababa) [27] and 20.40% (Harari) [34], respectively for HBV and 0.42% (Oromia) [17] and 4.30% (Amhara) [28], respectively for HCV.A total of 18 studies reported the prevalence of HBV among HCWs, thus the pooled prevalence of HBV using a random effect model was 5.93% (95% CI; 3.22–8.63) with no evidence of heterogeneity (I^2^ = 0.0%; p = 0.847) (**Fig 2**). Whereas, the pooled prevalence of HCV from 7 studies using a random effect model was 1.12% (95% CI; -4.19–6.43), no evidence of heterogeneity (I^2^ = 0.0%; p = 1.000) (**Fig 3**).

### Sub-group analysis

The prevalence of HBV and HCV among different types of sampling methods, types of the study population/group, study year, study setting/regions, and the types of diagnostic methods to detect HBsAg and/or anti-HCV were addressed using sub-group analysis by random effect model.The sub-group analysis by study population identified that the pooled prevalence of HBV among medical waste handlers and health professionals was8.6% (95% CI; 3.01–14.13) (I^2^ = 5.3%; p =

0.386) and 4.98% (95% CI; 1.85–8.11) (I^2^ = 0.0%; p = 0.968), respectively (**Table 2**).

**Table 2:** Subgroup analysis of hepatitis B virus prevalence among healthcare workers in Ethiopia.

Characteristics	Number of Studies	Sample size	HBsAg positive cases	The estimated pooled prevalence of HBV (95% CI)	Heterogeneity		
					I^2^ (%)	P-value	
**Study population**							
Health professionals	11	3723	182	4.98 (1.85–8.11)	0.0		0.968
Medical waste handlers	7	1225	100	8.6 (3.01–14.13)	5.3		0.386
**Sampling method**							
Simple random	5	1145	35	3.10 (-2.60–8.80)	0.0		0.970
Systematic random	1	126	8	6.35 (-10.55–23.25)	NA		NA
Convenient	10	2985	135	4.89 (1.39–8.39)	0.0		0.994
Multi-stage	2	692	95	14.60 (4.19–25.00)	54.7		0.137
**Study year**							
1987–2000	1	438	25	5.71 (-3.39–14.80)	NA		NA
2001–12	5	857	54	6.39 (-0.09.19–12.87)	0.0		0.943
2013–21	12	3653	203	5.84 (2.70–8.99)	0.0		0.495
**Study region**							
Amhara	5	1035	50	4.86 (-1.08–10.80)	0.0		0.990
Oromia	4	1419	59	4.20 (-0.89–9.30)	0.0		0.877
Addis Ababa	6	1650	76	4.70 (-0.01–9.41)	0.0		0.910
Harari	2	692	95	14.60 (4.19–25.00)	54.7		0.137
Sidama	1	152	2	1.32 (-14.48–17.11)	NA		NA
**Diagnostic method**							
Rapid test kit	7	1920	154	8.37 (3.63–13.10)	13.5		0.327
ELISA	10	2715	126	4.70 (1.03–8.38)	0.0		0.996
CIA	1	313	2	0.64 (-10.40–11.68)	NA		NA

HBsAg: hepatitis B surface antigen, ELISA: enzyme-linked immunosorbent assay, CIA: Chemiluminescent Immuno-assay NA: not applicable, CI: confidence interval.

This meta-analysis also found1.44% (95% CI; -5.28–8.18) (I^2^ = 0.0%; p = 0.999) and 0.59% (95% CI; -8.09–9.27) (I^2^ = 0.0%; p = 0.970) combined prevalence of HCV among medical waste handlers and health professionals, respectively (**Table 3**). Analysis of sub-group by study region identified that the highest prevalence of HBV was found among studies conducted in Harari region; 14.60% (95% CI; 4.19–25.00) with moderate heterogeneity (I^2^ = 54.7; p = 0.137) and the lowest HBV prevalence was found in a study conducted in Sidama region; 1.32% (95% CI; -14.48–17.11). Similarly, the highest prevalence of HCV was observed among studies conducted in the Amhara region; 1.62% (95% CI; -7.70–10.94) (I^2^ = 0.0%; p = 0.969) whereas, the lowest prevalence of HCV was found in a study done in Oromia region; 0.42% (95% CI; -12.21–13.04) (**Tables 2 and 3**).

**Table 3:** Subgroup analysis of hepatitis C virus prevalence among healthcare workers in Ethiopia.

Characteristics	Number of Studies	Sample size	Anti-HCV positive cases	The estimated pooled prevalence of HCV (95% CI)	Heterogeneity	
					I^2^ (%)	P-value
**Study population**						
Health professionals	2	507	3	0.59 (-8.09–9.27)	0.0	0.970
Medical waste handlers	5	839	12	1.44 (-5.28–8.18)	0.0	0.999
**Sampling method**						
Simple random	2	310	4	1.32 (-9.74–12.38)	0.0	0.772
Convenient	5	1036	11	1.06 (-4.99–7.12)	0.0	1.000
**Study year**						
2001–12	2	367	3	0.82 (-9.37–11.01)	0.0	0.983
2013–21	5	979	12	1.24 (-4.99–7.46)	0.0	0.999
**Study region**						
Amhara	3	435	7	1.62 (-7.70–10.94)	0.0	0.969
Oromia	1	240	1	0.42 (-12.21–13.04)	NA	NA
Addis Ababa	2	519	6	1.16 (-7.40–9.71)	0.0	0.924
Sidama	1	152	1	0.66 (-15.19–16.50)	NA	NA
**Diagnostic method**						
Rapid test kit	2	252	2	0.79 (11.50–13.09)	0.0	0.979
ELISA	5	1094	13	1.20 (-4.69–7.09)	0.0	0.999

Ant-HCV: anti-hepatitis C virus, ELISA: enzyme-linked immunosorbent assay, NA: not applicable, CI: confidence interval.

The highest combined prevalence of HBV per year of study was found among studies conducted between the years 2001 and 2012; 6.39% (95% CI; -0.09.19–12.87) (I^2^ = 0.0%; p = 0.943) and the lowest prevalence of HBV was observed in a study done before the year 2000; 5.71 (95% CI; -3.39–14.80). For HCV, the combined prevalence was 1.24% (95% CI; -4.99–7.46) (I^2^ = 0.0%; p = 0.999) and 0.82% (95% CI; -9.37–11.01) (I^2^ = 0.0%; p = 0.983) among studies conducted between the years 2001 to 2012 and 2013 to 2021, respectively (**Tables 2 and 3**).

This meta-analysis also performed sub-group analysis by the diagnostic methods used and identified that the pooled prevalence of HBV was found 8.37% (3.63–13.10) (I^2^ = 13.5%; p = 0.327), 4.70% (95% CI; 1.03–8.38) (I^2^ = 0.0%; p = 0.996), and 0.64% (95% CI; -10.40–11.68) among studies detected HBsAg using a rapid test kit, ELISA, and CIA, respectively (**Table 2**). Besides, the combined prevalence of HCV among studies that detected anti-HCV using ELISA and rapid test kit was 1.20% (95% CI; -4.69–7.09) (I^2^ = 0.0%; p = 0.999) and 0.79% (95% CI; -11.50–13.09) (I^2^ = 0.0%; p = 0.979), respectively (**Table 3**).

### Prevalence of HBV vaccination status among healthcare workers in Ethiopia

In this systematic review and meta-analysis study we estimated the pooled prevalence of HBV vaccination status among HCWs. Even though, all 18 studies included in this review had not reported complete data on the number of fully vaccinated HCWs; data from 9 studies showed that the prevalence of HBV full-dose vaccination status among HCWs was 14.62% (95% CI; -15.63–44.87) (I^2^ = 98.9%; p = < 0.001). The minimum and maximum prevalence of HBV vaccination status was 1.6% (Addis Ababa) (95% CI; -9.39–12.59) [27] and 83.33% (Harari) (95% CI; 79.48–87.18) [23], respectively (**S1 Fig**).

### Publication bias

The presence of publication bias was assessed statistically for the included studies using Egger’s statistics test at a significant level of less than 0.05. The findings of the Egger test revealed that there was no publication bias in both HBV (p = 0.846) and HCV (p = 0.12) (**Tables 4 and 5**).

**Table 4:** The Egger’s test to assess the publication bias on the prevalence of HBV among healthcare workers in Ethiopia.

Egger’s test					
Std_Eff	Coef.	Std.Err.	t	p-value	95% Confidence Interval
slope	6.78	4.51	1.50	0.152	-2.78–16.35
bias	-0.15	0.77	-0.20	0.846	-1.78–1.48

**Table 5:** The Egger’s test to assess the publication bias on the prevalence of HCV among healthcare workers in Ethiopia.

Egger’s test					
Std_Eff	Coef.	Std.Err.	t	p-value	95% Confidence Interval
slope	-1.12	1.40	-0.80	0.460	-4.73–2.48
bias	0.32	0.19	1.64	0.162	-0.18–0.82

**Fig 4:**
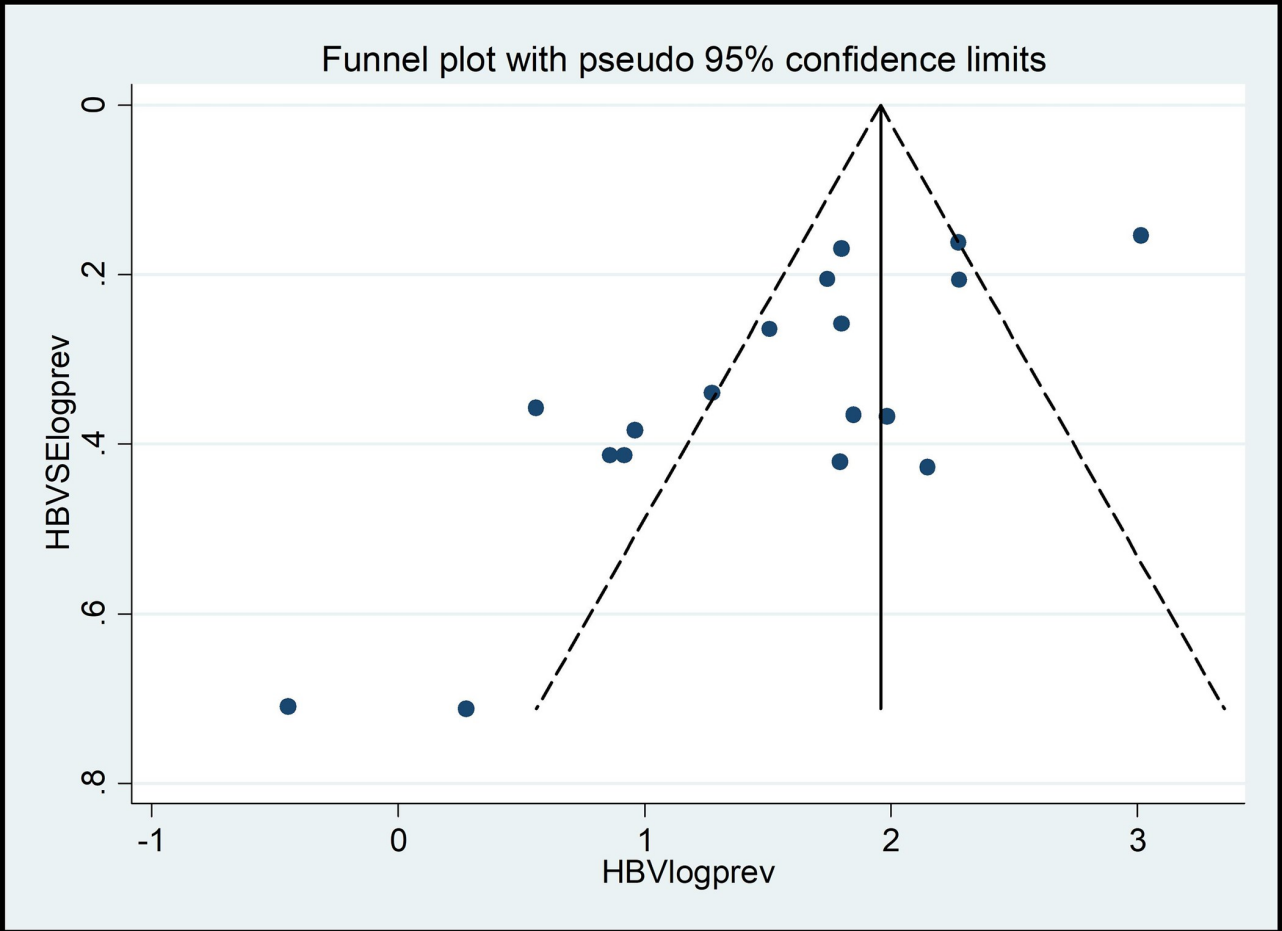
The funnel plot showing the publication bias of studies on the prevalence of HBV in Ethiopia.

**Fig 5:**
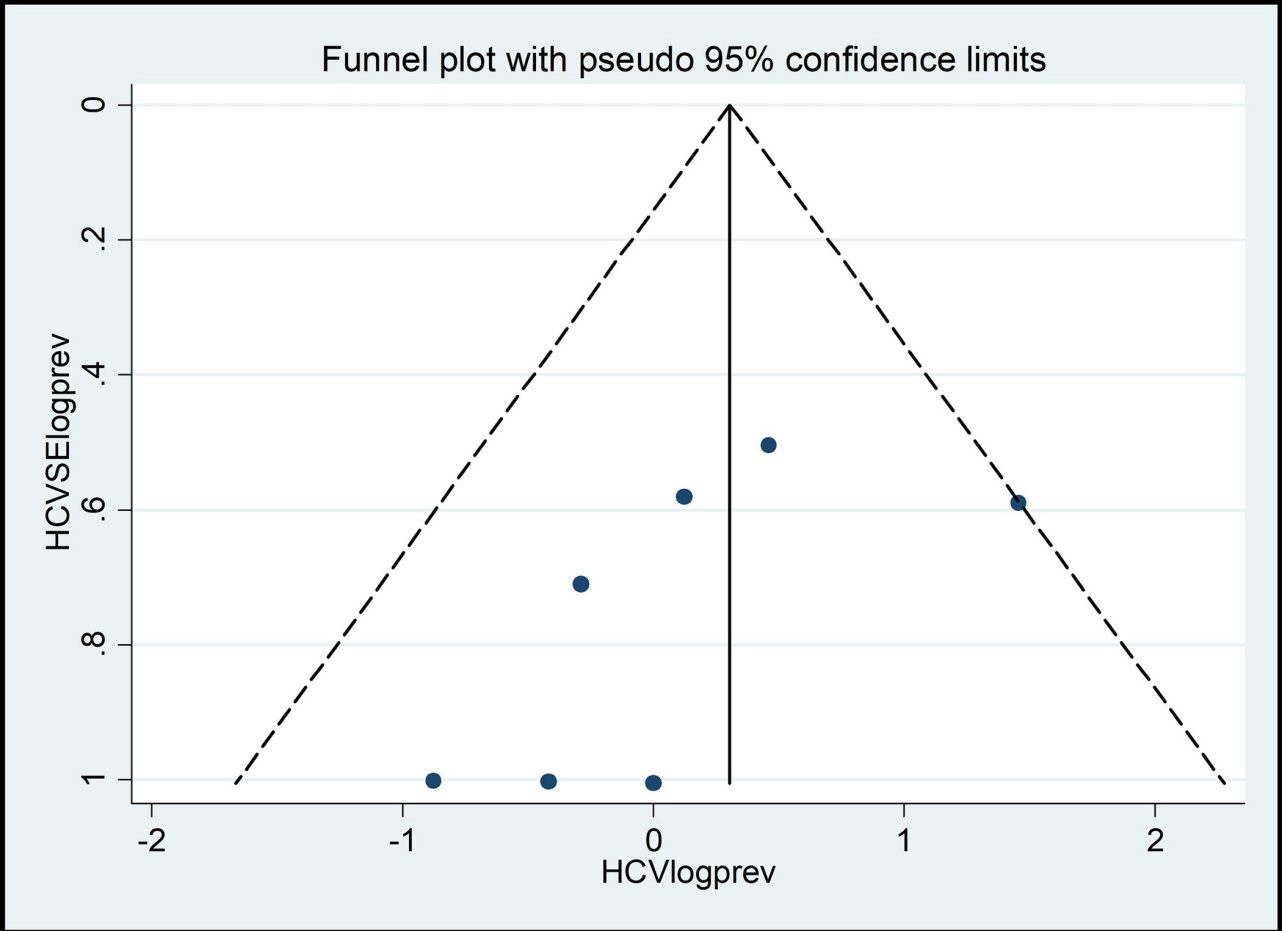
The funnel plot showing the publication bias of studies on the prevalence of HCV in Ethiopia.

These results were depicted using a funnel plot which showed a symmetrical display of the prevalence reported by all the included studies in this systematic review and meta-analysis (**Figs 4 and 5**).

## Discussion

In this systematic review and meta-analysis, we estimated the pooled prevalence of HBV and HCV infection among HCWs in Ethiopia. A total of 4,948 HCWs from 18 studies was used to determine the combined prevalence of HBV and/or HCV. Thus, the pooled prevalence of HBV and HCV was 5.93% (95% CI; 3.22–8.63) and 1.12% (95% CI; -4.19–6.43), respectively. The combined prevalence of HBV in this meta-analysis was almost similar to the previous meta-analysis conducted in Ethiopia [38] which reported 6% HBV pooled prevalence. The current prevalence of HBV was consistent with the WHO HBV infection endemicity classification with 5–7% of HBsAg prevalence [39] and comparable with the previous meta-analysis report from Ethiopia;7.4% [6]. Moreover, the prevalence of HBV in this study was also in line with previous meta-analysis studies conducted among HCWs from Africa; 6.81% [40], and Asia and Africa; 5% [41].

On the other hand, the pooled prevalence of HBV in this study was higher than previous global meta-analysis reports among HCWs and waste collectors [42–44], where the combined prevalence of HBV ranged from 0.04% to 2.3%. In addition, the prevalence of HBV in this study was higher compared with previous reports among HCWs from eastern Mediterranean and Middle Eastern countries [45] and Iranian healthcare workers [46] with an HBV pooled prevalence of 0.4% and 2.77%, respectively. These discrepancies in the variation of HBV prevalence might be due to the difference in the disease distribution among the developed and middle-income countries in the world [2, 47]. This variation might be also attributed to inadequate access to HBV screening and patient management, lack of HBV vaccination utility, and limited adherence to WHO safety recommendations among HCWs and medical waste collectors in developing countries [16, 48, 49]. Besides, the combined prevalence of HBV in this study was lower than previous reports from Sudan [50], Nigeria [51], and Vietnam [52], where the prevalence of HBV among the general population was 12.07%, 9.5%, and 10.5%, respectively. The current prevalence of HBV was also lower compared with a previous meta-analysis from Brazil among waste collectors [53]. The variation in the combined prevalence observed among different regions of the world might be due to the difference in study population (general population and/or waste collectors versus HCWs and/or medical waste handlers) and the difference in HBV diseases distribution.

Whereas, the current combined prevalence of HCV was comparable with previous Ethiopian meta-analysis studies [6, 54] which reported that the pooled prevalence of HCV in the country was 2% and 3.1%, respectively. The prevalence of HCV in this study was in line with previous meta-analysis reports from Vietnam [52] and Sudan [50], where the HCV prevalence reports were 0.26% and 2.74%, respectively. Moreover, our finding on the prevalence of HCV was comparable with previous Global reports among waste handlers; 0.08% [43] and African meta-analysis study among HCWs; 5.58% [40]. This variation in the prevalence of HCV across different regions might be due to the different rates of exposure to HCV among HCWs and/or medical waste handlers versus the general population. The possible reason for the discrepancies might be that HCWs and/or medical waste handlers are highly vulnerable to occupational-associated blood and body fluid exposure as compared to the general populations, where the major route of HCV transmission is through contact with blood and body fluids [55, 56].

The sub-group analysis on the prevalence of HBV among HCWs by the study population was observed in Ethiopia. Our finding showed that the combined prevalence of HBV was higher among medical waste handlers (8.6%) than health professionals (4.98%). Besides, the prevalence of HCV was almost comparable between health professionals (0.59%) and medical waste handlers (1.44%). Our finding was per the fact that medical waste handlers had a higher rate of exposure to sharp materials, blood products, and body fluids [57, 58]. This variation in the prevalence of HBV among medical waste handlers versus health professionals might be due to the difference in the HBV vaccination coverage, limited knowledge and awareness of medical waste handlers on the transmission of HBV and waste management, low adherence of medical waste handlers to the WHO safety recommendations [20, 59, 60]. The prevalence of HBV among HCWs in this study was observed indifferent regions of Ethiopia which showed that a higher combined prevalence of HBV was observed in the Harari region (14.60%)than Amhara region (4.86%), Addis Ababa city administration (4.70%), Oromia region (4.20%), and Sidama region (1.32%). Besides, the prevalence of HCV in this study was almost comparable across regions of Ethiopia, where the HCV prevalence was 1.62% in the Amhara region, 1.16% in Addis Ababa city administration, 0.66% in the Sidama region, and 0.42% in Oromia region. Accordingly, our findings showed that a higher prevalence of HBV was observed in the Harari region. This finding was supported by previous Ethiopian studies conducted in eastern Ethiopia which reported a higher burden of HBV among different population groups [61–63],where the HBV prevalence was 11.5%, 11.7%, and 7.85%, respectively. The prevalence of HBV among HCWs showed that there was a slight decrement across different categories of study years as it was 6.39% and 5.84% in studies conducted between the years 2001–12 and 2013–21, respectively. This slight decrement in the combined prevalence of HBV across study years might be due to a slightly increasing awareness of HBV transmission, diagnosis, and implementation of preventive medicines across years which was supported by the recent study done by Chonka et al. showed that the majority of study participants had a good knowledge to HBV infection [64]. The combined prevalence of HBV among HCWs by rapid test kit and ELISA was 8.37% and 4.70%, respectively. Accordingly, the result suggested that a higher prevalence of HBV was observed by rapid test kits than by ELISA tests. Even though, rapid test kits are widely employed in developing countries to screen HBV and HCV infections [65]. The higher prevalence of HBV by rapid test kits in this meta-analysis was supported by a previous cohort study conducted in France by Julie et al. which showed that rapid tests could produce a false negative result and a limited positive predictive value [66].

In this systematic review and meta-analysis study we estimated the pooled prevalence of HBV full-dose vaccination status among HCWs. Data from 9 studies showed that the prevalence of HBV full-dose vaccination status was 14.62%. Accordingly, results suggested that the prevalence of fully vaccinated HCWs was still low. Our finding was supported by previous meta-analysis studies [16, 67] which showed that the prevalence of full-dose HBV vaccine coverage among HCWs was 20.04% and 24.7%, respectively. This variation in the prevalence of HBV vaccine coverage might be attributed to the lack of cost-effective strategies that maximize the benefit of HBV vaccination to HCWs and limited access and utilization of HBV vaccine [68].

As strength, this study strictly adhered to the PRISMA guidelines and applied a comprehensive literature search, quality assessment, and data extraction from relevant articles by two independent authors, and discrepancies were resolved by the third author. In addition, we included all studies conducted in Ethiopia from December 1989 to April 2024which helps to generalize the pooled prevalence of HBV and HCV infection in the country. However, as a limitation, this study included some studies containing small sample sizes, which might affect the combined prevalence of HBV and HCV infection among HCWs in the country.

## Conclusion and recommendations

In this review, we found a higher and moderate prevalence of HBV and HCV infections, respectively among Ethiopian HCWs, particularly among Health professionals and medical waste handlers. Thus, strict adherence to infection control measures, effective implementation of prevention and control policies for HBV and HCV infections. In addition, adequate HBV vaccination coverage for HCWs is needed to reduce the burden of HBV infection in the country.

## Supporting information

S1 Table Showing the Preferred Reporting Items for systematic review and meta-analysis (PRISMA) 2020 checklist. (DOCX)

S2 Table Quality assessment of individual studies included for meta-analysis on the prevalence of HBV and HCV among healthcare workers in Ethiopia. (DOCX)

S1 Fig The forest plot showing the pooled prevalence of HBV vaccination status among healthcare workers in Ethiopia. (TIF)

S1 File Dataset used for analysis. (RAR)

## Abbreviations

Anti-HCV: Antibody to Hepatitis C Virus
CIA: Chemiluminescent immunoassay
ELISA: Enzyme-linked immunosorbent assay
HBsAg: Hepatitis B surface antigen
HBV: Hepatitis B Virus
HCV: Hepatitis C Virus
HCWs: Healthcare workers
PRISMA: Preferred Reporting Items for Systematic Reviews and Meta-Analyses
WHO: World Health Organization
