# Supplementary material for: Seroprevalence of viral hepatitis B and C infections among healthcare workers in Ethiopia: A systematic review and meta-analysis
Source: PLoS One. 2024 Nov 7;19(11):e0312959. doi: 10.1371/journal.pone.0312959 (PMC11542802; doi:10.1371/journal.pone.0312959)
Supplement: S2 Table — (DOCX) [file pone.0312959.s002.docx]

**S2 Table. Quality assessment of individual studies included for meta-analysis on the prevalence of HBV and HCV among healthcare workers in Ethiopia**

| **Author, year** | **Q1** | **Q2** | **Q3** | **Q4** | **Q5** | **Q6** | **Q7** | **Q8** | **Q9** | **Total score** | **Quality of the study** |
| --- | --- | --- | --- | --- | --- | --- | --- | --- | --- | --- | --- |
| Desalegn&G/Selassie, 2013 | 1 | 0 | 0 | 0 | 0 | 1 | 1 | 0 | 1 | 4 | Moderate |
| Geberemicheal et al, 2013 | 1 | 1 | 0 | 1 | 0 | 1 | 1 | 0 | 0 | 5 | Moderate |
| Kefenie et al, 1989 | 1 | 0 | 1 | 1 | 0 | 0 | 0 | 0 | 1 | 4 | Moderate |
| Abate et al, 2022 | 1 | 1 | 1 | 1 | 0 | 1 | 1 | 1 | 0 | 7 | High |
| Yizengaw et al, 2018 | 1 | 1 | 1 | 1 | 0 | 1 | 0 | 1 | 1 | 7 | High |
| Akalu et al, 2016 | 1 | 0 | 0 | 1 | 0 | 1 | 1 | 1 | 1 | 7 | High |
| Gebremariam et al, 2019 | 1 | 0 | 0 | 1 | 0 | 1 | 1 | 0 | 1 | 5 | Moderate |
| Yilma et al, 2021 | 1 | 1 | 1 | 1 | 0 | 1 | 1 | 1 | 1 | 8 | Higsh |
| Seid et al, 2005 | 1 | 0 | 0 | 1 | 0 | 1 | 0 | 1 | 1 | 5 | Moderate |
| Tufa et al, 2016 | 1 | 0 | 1 | 1 | 0 | 1 | 0 | 1 | 1 | 6 | Moderate |
| Hebo et al, 2019 | 1 | 1 | 0 | 1 | 0 | 0 | 1 | 0 | 0 | 4 | Moderate |
| Amsalu et al, 2016 | 1 | 0 | 0 | 1 | 0 | 1 | 1 | 1 | 0 | 5 | Moderate |
| Mengiste et al, 2021 | 1 | 1 | 0 | 1 | 0 | 1 | 1 | 1 | 0 | 6 | Moderate |
| Shiferaw et al, 2011 | 1 | 1 | 0 | 1 | 0 | 0 | 0 | 1 | 1 | 5 | Moderate |
| Mussa et al, 2022 | 1 | 0 | 1 | 1 | 0 | 0 | 0 | 1 | 1 | 5 | Moderate |
| Anagaw et al, 2012 | 1 | 0 | 0 | 1 | 0 | 0 | 1 | 0 | 1 | 4 | Moderate |
| Ayele et al, 2023 | 1 | 1 | 0 | 1 | 0 | 0 | 1 | 1 | 1 | 6 | Moderate |
| Mekonnen et al, 2015 | 1 | 1 | 0 | 0 | 0 | 1 | 0 | 1 | 1 | 5 | Moderate |

1: for “Yes”, 0: for “Not reported” or “Not appropriate”.

The quality of studies was classified as low (total score; 0 to 3), moderate (total score; 4 to 6), and high (total score; 7 to 9)

Q1 = was the sample frame appropriate to address the target population?, Q2 = were study participants sampled in an appropriate way?, Q3 = was the sample size adequate?, Q4 = were the study subjects and the setting described in detail?, Q5 = was the data analysis conducted with sufficient coverage of the identified sample?, Q6 = were valid methods used for the identification of the condition?, Q7 = was the condition measured in a standard, reliable way for all participants?, Q8 = was there an appropriate statistical analysis?, Q9 = was the response rate adequate, and if not, was the low response rate managed appropriately?
