# Supplementary figures and images for: Seroprevalence of viral hepatitis B and C infections among healthcare workers in Ethiopia: A systematic review and meta-analysis
Source: PLoS One. 2024 Nov 7;19(11):e0312959. doi: 10.1371/journal.pone.0312959 (PMC11542802; doi:10.1371/journal.pone.0312959)

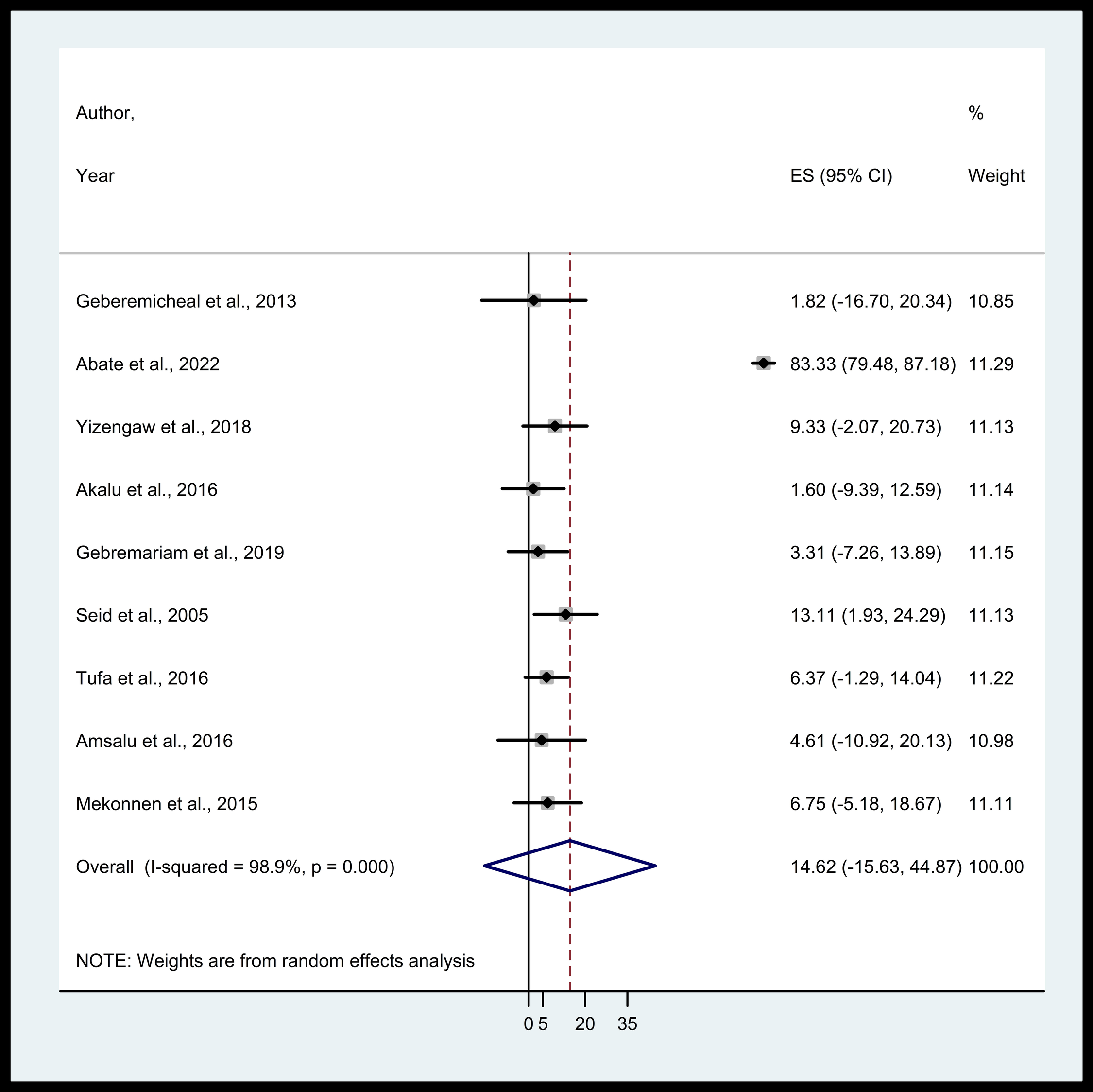

Supplement: S1 Fig — (TIF) [file pone.0312959.s003.tif]
